# Extracts

**Published:** 1883-11

**Authors:** 


### Extracts.

IMPOTENCE AND NOCTURNAL EMISSIONS, TREATED BY GALVANISM. — Perhaps a majority, certainly a large proportion of cases consulting a neurologist in a given length of time, are the outcome of generative insufficieucy. No disease, if indeed the hydra-headed group of disorders of mind, muscle and nerve arising from this cause may be so termed, is so wide spread, so generally understood to exist or so rarely leads to a fatal termination as this, and we may add, is so unsuccessfully treated. In the nature of things, this condition has probably always existed, will always remain. When the sexual instinct first makes its appearance in the pubescent boy or girl, there accompanies the development of its strong desires, a demand for its gratification so peremptory that it is rarely unheeded, and attains its satisfaction either in waking or in sleeping dreams. The number of men who attain adult life without masturbation, is surprisingly small, and is, I am persuaded, confined to those who have had early and constant intercourse with women, or who are arrayed in the ranks of nocturnal emissionists.

If a general inquiry were to be instituted amongst men of, say, twenty-four years of age, as to how many *never* have masturbated, the result would astonish, not the experienced medical man, who knows the facts, but nearly every one else, and might perhaps, be doubted. None the less true, however.

The sexual appetite is one which will not be denied gratification when it comes upon a young man, and is scarcely less imperious than its fellow instinct of life preservation. The habit of sel-abuse is contracted, in the male, from companions almost exclusively, and usually persists until the boy grows large enough to go into female society, where he speedily becomes inducted into the knowledge of genuine sexual pleasure, and abandons the old habit with disgust. So that impotence from masturbation is by no means as common as is generally supposed, but is usually limited to the class before alluded to as exceptional, — those constantly with women or haunted by emissions.

Not only in the male; for I am constantly consulted by women who are as useless in sexual matters as an eunuch. They can neither receive nor give sexual delights — know nothing of the strong desire for male companionship which is a normal instinct, and, if conscientious persons, who have unwittingly entered the married state, are in life-long discomfort, not to say misery. "Doctor," asked one, "what shall I do? My husband tells me that I am good for nothing, that he never saw such an iceberg, and that I must consult you in the matter. For, if there is any cure, I must have it, or he may not remain true." Alas, poor lady, there is none. You were born so in common with many others of your sex, and to attempt to develope an instinct that only exists in a rudimentary condition is well nigh a hopeless task.

So general is this condition of sexual apathy amongst women of all nations, that to find one whose desires are as strong as those of the average man is the exception to a well-known rule; and were this as generally understood by the laity as by the profession, it is possible that much domestic infelicity might be avoided. Not to expect too much is to avoid disappointment.

While masturbation is much less common with girls than with boys, it still exists to a degree that would astonish anyone ignorant upon the subject.

We are not so badly off as in France, if an eminent author is to be believed. Dr. Deslandes states that "a large number of little girls and majority of adolescents are addicted to this habit." But there is enough of it with us, and it is the parent of many of the hysteriform nerve derangements of our young women. So marked is the peculiar expression of one of this class, that one almost never makes a mistake in asking, during an examination, the plain question, "How many times a week do you practice self-abuse?" The reply, made with downcast eyes and blushing cheeks, "Why, doctor, how could you know that?" and a little judicious coaxing soon makes the matter clear, and there is no trouble with the diagnosis.

In the treatment of these cases, more men have grown rich than in California gold mines — for the pride of man is his virility, and he will part with anything short of life rather than lose it. So that, in all ages charlatans have reaped a rich harvest from this field, and the nineteenth century still gives them a plentiful aftermath, for it is one wherein regular medicine has made such brilliant failures that physicians are chary of promises.

Damiana, phosphorus, strychnia, musk, nutmeg, and the various preparations of iron have all been faithfully tried as aphrodisiacs and, with the exception of the first-named, have been of greater or less benefit in selected cases. Damiana has, in my hands, proven as useless as cundurango in cancer, and it is, I think, very generally discarded. The others are most valuable as general tonics, with perhaps a special remedial action in generative insufficiency, but I have never seen, outside of the Grand Bazaar at Constantinople, any drug which was a direct and undoubted aphrodisiac. There was a crimson elixir sold there a few years since which was certainly that — and which acted without any subsequent loss of power, so far as a limited experience of it warrants me in speaking, but its composition was entirely a secret.

Electricity has been employed for a long time in these cases, until quite lately by charlatans alone, and even in their hands has yielded good results. It is to call attention to a few points which are perhaps novel in the application of the currents to the generative organs, that this article is written, although the long preamble might easily have led to another opinion. Early in my special study of electro-therapeutics, in 1874, the idea occurred to me that faradism could be applied to the male generative organs in a better way than by manipulation with rheophoeres or the electric brush. I therefore devised a special electrode, in the shape of a cylinder of metal within which plays a piston, the rod of which is surrounded by a weak spiral spring bearing upon the inside of the closed end of the cylinder. Resting upon the piston is a pad of surgeon's sponge. The tubes are made of different diameters varying from three quarters to two inches, and from three and one-half to five inches in length; and, during the application, surround the penis with the sponge-pad moistened with salt water pressing against the glans. The current should be exceedingly fine faradism; i. e., of rapid interruption, of high tension, and fair quantity. In other words. it should be as strong as the patient can bear without pain and without shocks. Applied in this way, with a large negative sponge electrode at the back, under the lumbar vertebræ, the pat ent comfortably recumbent, the result is a strong stimulation of those branches of the sacral plexus composing the genito-urinary tract and a corresponding increase in muscle nutrition. The penis becomes turgid, the dartos contracted, and a close watch must be kept upon the patient, lest by continuing the application too long, an emission be produced. By discontinuing the current after five minutes, or by increasing the olectro-motive force until it becomes painful, this result may be avoided. By a steady persistence for several months in this form of treatment, I have succeeded in rescuing several patients from that worse than death to a sensitive man, — a life of lost manhood. The applications should be made daily.

In cases of nocturnal emissions, a directly contrary course must be pursued, since the conditions are opposite, at least in a certain sense. There is as yet no loss of virile power—there is an intense hyperæsthesia of the genito-urinary tract, which requires no stimulus, which is directly injured by it. No tonics, beyond the general ones of out-door exercise and plentiful food, can be depended upon; for they tend to aggravate the irritation of the excited nerve centers. For this reason, faradism is inadmissible. I think that far more injury than good has followed its use, and abandoned it wholly in my own practice in these cases several years since. In its place I use galvanism, derived from a battery of low tension—some modification of Daniell's element. It is applied by a Newman's urethal electrode, with olive tip large enough to gently distend the urethra. This is passed into the bladder, and the circuit completed through a hand electrode, when it is slowly withdrawn, the whole operation lasting about one minute. If the slightest pain is experienced, the current is too strong, and should be modified until only a sensation of warmth is perceived. This operation should be repeated daily for several weeks, and has been in my practice productive of the happiest results. One case, now under treatment in the eighth week, reports emissions reduced from three to four weekly to one in eight or ten days.

What can electricity do for apathetic women? I regret to reply, very little. Years of work in this direction have convinced me that no agent can overcome a natural lack of sexual passion. Faradism, general and ovarian; galvanism, spinal and central; franklinism in every form of application, have each been faithfully tried and in turn have failed to effect any permanent good. If the general health be good, I now recommend consulting women to abstain from all treatment for such condition and rest content with their natural status.

There are, however, occasional cases wherein there is a slowness of action of temperament; where the passions are sluggish and torpid, which electricity is competent to remedy. Here spinal and ovarian faradization has proven most useful.

I cannot close this article without calling attention, in the strongest possible manner, to a point wherein I think the profession is strangely, culpably derelict. We do not insist sufficiently upon the necessity of home training. If every father and mother would talk plainly to their boys or girls, would explain to them the danger of secret practices and how difficult to get rid of the habit once formed—if family physicians would insist upon such confidences between parents and children, much misery and shame might be averted. In every case that comes under my care I ask, "Did your parents never tell you of the danger of self abuse, and warn you against contracting the habit?" And invariably the reply is "No indeed." The influence of the house doctor in cultivated families—in all families—is greater than any one but himself can estimate, and his clients have the right, if it be an unsuspected one, that he shall use *all* his talents in their behalf.—*William F. Hutchinson, in New England Medical Monthly.*

---

THE THERAPEUTICS OF BLUFF.—Dr. E. P. Hurd, in a recent editorial article in the *Medical Record*, gives some very common sense suggestions touching the conduct of the physician at the bedside. He divides practitioners, as regards their manner in the sick-room, into two classes, a representative of each of which he follows on his rounds. Dr. Blank is of the highly conscientious, very scientific and hesitating type of physician. He is attending Mrs. Brown, an elderly lady, who has extensive consolidation of the lung. He makes a very careful examination of the thorax with his Cammann binaural stethoscope, and arrives at a very gloomy prognosis. Although not informing the [family that the old lady will die, his prognosis is written so clearly on his face that they who run might read it there. His whole conduct and deportment convince the family rather of his inability to comprehend or treat the case than of the patient's serious condition. Under these circumstances a change of doctors is determined upon, and Dr. Bluff is called in. Bluff is in no sense a scientific man, nor does he make any great pretentions in that direction, priding himself, doubtless, on some innate and indescribable power of penetrating the mysteries of disease rather than on his medical knowledge. He is a man of the world, drives fast horses, and is "hail fellow well met." His cheery manner has caused people to say that they would rather have

Bluff's presence in a sick room, even though he did nothing more than talk slang, than that of the most scientific college professor, who would talk learnedly, prescribe nauseous medicine, and tell them of tho gravity of their disease.

Bluff was ushered into the room of the sick Mrs. Brown. After listening to the diagnosis and fearful prognostications which poor Dr. Blank was reported to have made, he turns them all into ridicule. "Nonsense," he says, "only a little stuffing in the chest," which he would "clear out in less than no time." He calls the old lady "a daisy," "a blooming rose of Sharon," "a gay old girl," who has "not got through her sparking yet," and ventures the remark, "that if the present Mrs. Bluff should ever be taken off, he would improve his opportunity, etc. As for dying, "fiddle sticks! she cannot die with *that* pulse, and I will have her out of that bed, scrubbing the kitchen floor, before a week." The whole result of this talk is to kindle hope, and to inspire confidence on the part of the Browns in Bluff's ability. The patient herself is even buoyed by it, and apparently improves under the change of treatment. The improvement, however, does not last long. The inevitable progress of the disease is unchecked, and in due time Dr. Blank's prognosis is verified. The effect of Dr. Bluff's manner has, however, not been lost on the Brown family, and recalling the apparent improvement which followed the first few day's of Bluff's treatment, they will always believe that had they called him in before the disease had gone too far, their venerable and now sainted mother would have been spared to them.

This picture is by no means a purely imaginary one. Dr. Blank and Dr. Bluff are the prototypes of many men who honor or dishonor our noble profession. The latter will generally be the most popular, if not the most successful. While by no meansendorsing Bluff's style, there is no room to doubt the fact that encouraging words and smiles are often of real therapeutic value.—*Therapeutic Gazette.*

---

THE TREATMENT OF ACUTE RHEUMATISM.—The remedies which have, from time to time, been recommended in the treatment of acute rheumatism embrace, probably, the larger percentage of the drugs with which physicians are most familiar. A remedy which will prove useful in one case will turn out to be absolutely useless in another. This fact has been very generally noticed, and, as a consequence, the disease has been one of the most perplexing which the physician is called upon to treat. Anything which may tend to explain the discrepancies in the effects of different agents in the treatment of disease as occurring in different persons, will be very gladly welcomed by many a distressed and perplexed practitioner.

Dr. Robert Bartholow has, we think, recently given an analysis of rheumatism as occurring in different persons, which will prove valuable as an aid to the selection of drugs. He divides rheumatism into three different classes, according to the physical condition of the person in whom it occurs:

1. Those of considerable bodily vigor, spare habit, good muscular development, and having a distinct family history of neurotic or rheumatismal disorders.

2. Obese subjects, addicted to malt liquors and good living, with, but sometimes without, an inherited predisposition to rheumatic diseases—a class whom he graphically describes as "the gelatinous descendants of albuminous parents."

3. Pale, feeble, anæmic subjects, depressed by poor diet and bad hygienic surroundings, including dampness and bad air

He declares that no one can intelligently treat rheumatism without recognizing its type, as occurring in these three classes of subjects. He divides his remedies according to these classes. In the first class he administers salicylic acid or salicylate of soda. In this class there is a decided tendeney to relapses, and the salicylates must be continued for several days after all the acute symptoms have subsided.

In the second class the alkaline treatment is the one to be commended. By alkaline treatment, however, he understands something different from what is generally understood by such treatment. He adopts as his guide rules laid down by Dr. Fuller. It was Dr. Fuller's practice to give not less than 1½ drachm doses of the alkaline carbonates, either alone or in combination with a vegetable acid, during the first 24 hours of treatment. More commonly 2 drachms are ordered to be taken in effervescence, every three or four hours, in combination with an ounce of lemon juice, or with half a drachm of citric acid dissolved in four ounces of water. As soon as the urine, when freshly voided, ceases to show an acid reaction, which is usually the case after 24 hours, the quantity of the alkali is diminished by one half.

In the third type of rheumatic cases, those which are numerically the most important, the remedy *par excellence* is the tincture of the chloride of iron. To be effective this preparation must be given in full doses, from half a drachm to one drachm, in a sufficient quantity of water, every four to eight hours.

He attaches considerable importance to the application of blisters, which may be combined in all of the three classes of cases with the drugs recomended in each form. He makes no mention of the more recent form of the application of blister, namely, one four by four inches in size, placed immediately over the heart. We have had no personal experience in this treatment, but we call attention to it as the more recent method of the application of the blister treatment. It is said to be followed by a prompt reduction of the fever, and to be a very valuable prophylactic against the occurrence of cardiac complications.

The above suggestions may be valuable in determining the efficacy of manaca in the treatment of rheumatism. The reports which have been received bearing upon the value of this drug in rheumatism have been somewhat contradictory. While the majority of them attest the great value of the drug, not an inconsiderable number declare it to be inert as far as any relief of the diseased process is concerned. We would suggest that our readers, in their observations touching the value of this newer drug, will have reference to Dr. Rartholow's classification.

By so doing they may be able to establish definitely the class of cases in which manaca may be given with the hope of immediate benefit.

---

Convallaria Majalis in Heart Disease.—Recently efforts have been made to give this remedy the place of digitalis as a diuretic and remedy for certain forms of heart disease.

It is an old remedy. Culpepper regarded it as a valuable remedy for weak memory, lost speech and apoplexy. Gerarde recommended it for gout. For long years the peasants of eastern Europe have valued it in cases of dropsy. In 1880 Drs. Troitsky and Bogojavlensky, two Russian physicians, on investigating its action, said that it was valuable in certain forms of heart disease. Prof. Botkin, of St. Petersburg, confirmed most of these results. In July, 1882, Professor Germain See published the results of his experiments. (*Bull. Gen. Ther.*, *Brit. Med. Jour.*)

In 1858 Walz isolated two glucosides, which he named "convallarin" and "convallamarin." The investigation of their chemical and physiological properties by Tanret and Marme soon followed. It was found that convallarin possesses purgative properties only, while convallamarin is a heart poison allied to digitalis, helleborin, etc. The preparations usually employed are the aqueous extract of the leaves, an aqueous extract of the flowers and the extract of the entire plant. The last is the best for the obtaining of the full therapeutic effect.

A drop of the extract of the flowers injected under the skin of a frog arrests its heart in systole, very much as digitalis and some other remedies do. Four drops of this injected into the vein of a dog caused death in ten minutes. The heart appears to be first slowed, and the respirations are quickened. Then the heart's action becomes irregular and the pulsations weak and very rapid. The blood pressure first rises and then falls. The respirations gradually diminish. The heart stops first, then the pressure falls to zero, and the respiratory movements stop. The excitability of the pneumogastric is weakened, although not abolished

Prof. See reports five cases of mitral insufficiency characterized by want of rhythm, œdema of the lower extremities, dyspnœa, etc. The doses of the extract given were from seven to fifteen grains daily. In each case there was marked improvement; the heart's action becoming stronger, the breathing better and an increase in the amount of urine passed.

A case with mitral stenosis was also beneffted; so also several cases of aortic insufficiency.

Thus it appears that the favorable effects of this drug upon the heart and blood vessels are constant and reliable.

Favorable reports have been made as to its practical value in cases of palpitation from exhaustion of the pneumogastric, in simple cardiac erythema with or without hypertrophy, and with or without valvular lesions, in dilatation of the heart, etc.

Some observers have failed to get any appreciable effect from this drug. But it would seem from the mass of favorable evidence adduced that they must have either had a poor artlcle, or failed to use it in appropriate doses. —*Detroit Lancet*, May, 1883.

---

Suburban Homes.—(*R. S. Guernsey in the Sanitarian*)—The inspection of almost any of the suburban houses near large cities and towns in the United States as well as in Europe, will show an entire absence of sanitary knowledge or foresight on the part of the architect—by architect we mean any person who directs or superintends the planning or erection of a building. But generally, the larger and more imposing the structure, the less sanitary knowledge or care for it is shown. If any knowledge or instinct in regard to it appears, its effect is in the negative, by boldly violating the plainest rules of temperature, ventilation, pure air, and proper light, for the good health and happiness of the household, as well as a disregard for every-day conveniences of home life. These are all swept away by the architect for the purpose of giving outside "effect" to the structure and its surroundings, for a few months in the summer.

We do not now refer to the hotels and boarding houses of summer resorts, but merely to private summer residences, erected by the wealthy, and for luxury and health of the owner. If an architect is consulted about the form or plan of a proposed suburban residence, he first wishes to know whether it is on a hill, and how far from the street, and the size of the plot, and which side of the street or road it is, and the price of the intended structure. Not a word is asked about the location of the rooms for sleeping, or for other purposes. Nothing about draining, or the proximity of standing water, or swamps, or any noisome vapers from any source. The course of the summer prevailing winds are not heeded or taken into consideration.

The "effect" which the architect aims at is only that which may appear to be beautiful to the outside observer; but it is that in which perhaps not one person in ten thousand who may chance to see it ever observes or notices, as it is too fine for every day observation of passers-by. To one who has knowledge of sanitary laws, its surroundings make it appear to be a mere death-trap. It may be fair without, like the whited sepulchres of old, but full of death and noisome vapors within. When we get into this structure, which is so imposing or pretty from the outside, what are we to find? Rich and costly furniture, paintings and books, of coure; and we are just so sure of finding death dealing vapors, the prevailing air which must be breathed by day or night by those who stay there. We will not speak now of the almost studious absence of the convenient arrangement of rooms desired for any purpose of daily use. Plumbers have of late been very much blamed for their unsanitary work, but the real responsibility lies upon the architect; he has the shaping and directing of the usual supply and discharge pipes, and the only injunction that he seems to regard about them is, "that water will run down hill;" but where there is no hill for it to run down, he does not seem to know that it will stand still, or be absorbed by the atmosphere, and when it is saturated with filth, the atmosphere conveys its poisons into the bodies of those who breathe it, with more deadly effects than in any other manner.

This beautiful structure of a house is in reality a cover for a cesspool, and the waste-pipes convey its vapors to the sitting-rooms and sleeping apartments, or it floats in the open window of the bed-room on the cool breeze that the unconscious sleeper is inhaling at every breath for many hours every night, and the morning air is none the less from it. Royal families in Europe are suffering in this way to-day.

After awhile—it may be a few months or a few years—death steals away members of that household, and it becomes desolate, and is finally abandoned, and left in charge of a keeper, who occupies the most healthy part of the premises—being an outhouse of very simple construction.

Architects are to blame for this. No person wishes to have an unhealthy dwelling-place, and if any desire to build, they would sooner seek out a person that would plan them a convenient, healthy and to those who are to dwell within it, as well as only pretty from the outside, and where, in truth, distance only would make it appear beautiful.

Who can answer the question of where shall we find such an architect?

This is a field which cannot long remain idle, with the present knowledge and heed of sanitary laws.

---

WHAT SURGERY CAN DO.—The London *Lancet*, in publishing its record of the progress of medicine in its many departments during the last year, gave some of the more prominent points connected with surgery. Some of the operations seemed almost miraculous, and were regarded as impossible previous to experiment. No region of the body is now considered beyond the scope of surgery. Its most marked triumphs relate to the internal organs and cavities.

What has rendered the operations comparatively safe is the use of antiseptics—fluids that prevent putrefaction in the wounds. Hitherto carbolic acid has been the chief agent used. But this proved more or less dangerous—sometimes fatally so—in other directions. A much safer and equally effective substitute has been found in what is called eucalyptol, which is obtained from the eucalyptus tree.

Abscesses of the liver have been freely and successfully cut into and drained. Large parts of the stomach have been cut out, including even the pylorus, which is the more highly organized part of the stomach that shuts in the food until digestion is carried to a certain extent, and then opens and pours it into the intestines.

Entrances have been made through the walls of the stomach for the regular introduction of food in cases where the gullet has been closed by disease.

Two pieces embracing the entire circumference, the one about three inches in length, the other five, have been cut from the large intestine—the colon. In all cases the divided parts are brought together and sewed, the stitches becoming soon absorbed after the healing is complete.

Considerable progress has been made towards ascertaining the exact spot where the brain and nervous system are affected, thus facilitating the reaching of disease.

It has been found that bone can be transplanted and aid in the formation of new bone; and more wonderful still, that sponges can be grafted into a large wound, and be a porous support for the granulations—the new flesh particles—while they are filling the cavities. The sponge is believed to be gradually absorbed.

---

THE USE AND ABUSE OF BATHING.—Dr. Dudley A. Sargent, medical director of the Union gymnasium, gave the fourth of his talks of physical training at the Union hall last week, taking for his subject "Bathing; Its Uses and Abuses." He gave general rules for bathing as follows: "A warm bath, with liberal use of castile soap, is best for cleanliness, and night the best time. Twice a week is often enough. Too frequent warm baths debilitate the system. A cool sponge or wet cloth bath should be taken daily for its tonic effect, and always in a warm room. If strong and vigorous the best time is the morning; if not strong the cold bath had better be omitted and the tepid substituted. After exercise, if greatly fatigued, take no bath, but rub down vigorously with a dry towel. If thoroughly warmed up but not tired, take a tepid sponge bath standing. Never take a tub bath, except when bathing for cleanliness. A warm shower bath, followed by a cool sprinkling, is preferable to a cold bath after exercise. Vigorous exercise renders Turkish and hot baths unnecessary; those should be reserved for medical cases. Skin disorders are frequently caused by excessive bathing and the use of too much soap. Although no general rules for bathing could be given, every man must be guided by his own physical condition and his occupation.

---

BYRON'S DEATH, preceded by epileptic seizures and exposure to malaria, was hurried by what would to-day be the extremest malpractice. For five weeks, by his own rule, he had been living on toast and tea. At last, by the persistent urging of two doctors, he consented to be bled. (Date, April 16, 1824). They took *twenty* ounces. The next day they repeated the bleeding twice, and put blisters above his knees (he objected to having his feet exposed). The next day he was given two strong narcotic draughts, and the next day he died—and but little wonder. In this day such practice looks strange to us. Surely we have lived and learned.

---

HEAVY DAMAGES AGAINST A CHEMIST.—An action was recently brought against an Aberdeen druggist by the widow of a man who was alleged to have died through the negligence of one of the druggist's shopmen in supplying some poisonous compound by mistake for salicylate of soda. The case did not go to proof, but was settled by the defender paying £400 in full of the damages and the expenses sued for. A case such as this should impress on dispensing chemists the importance of making themselves thoroughly sure as to the qualifications and reliability of the assistants they engage in connection with their business.

---

THE following language is used by Dr. G. G. Budford (*N. O. Med. and Surg. Jour.*) in regard to the employment of alcohol in pneumonia: "Give your patient plenty of alcoholic stimulants, varied as occasion demands. Give it with the food. Give it with the liquids imbibed. Give it at regular intervals, and in doses to suit the age of the patient. Give that form that you know contains a constant quantity of alcohol, and the most nourishment. Then, with proper hygienic surroundings, you come nearest following out the indications of nature, and gain the best possible results."

---

Recent experiments made by the physiologist, Paul Bert, go to show that hydrophobia is not caused by the canine saliva, but by the phlegm contained in the bronchial tubes, which may or may not enter the wound together with the saliva. This explains why the bite of a rabid dog is not necessarily fatal. By filtering the foam which forms at the mouth of the dog it has been possible to separate the saliva from the phlegm. The latter, injected into an animal organism, infallibly produces hydrophobia, while the former does not

---

Eczema of the Scalp in Infants.—Dr. Lassar (*Gaz. Med.*) employs the following formula: Salicylic acid one, tincture of benzoin two, and vaseline fifty parts. A certain quantity of this is smeared over the scalp two or three times a day, after having washed the infant's head with soap and water. To soften the crusts and facilitate the cleansing of the scalp, Dr. Lassar recommends the employment of oil containing two per cent. of salicylic acid.

---

On the subject of blistering, Dr. Goodell says in the *Philadelphia Medical Times:* "I always use the cantharidal collodion. I shall paint a blister in this instance three by four inches, putting on three or four layers, and then at once put over this a poultice. This is an almost painless way of raising a blister. I have never seen it produce strangury."

---

Hot Water in Epistaxis.—Auquier mentions a case in which he was called to a man of twenty who had been suffering for three hours from violent epistaxis. The patient had been subject to such attacks from infancy. M. Auquier tried in vain to stop the bleeding by means of cold water.

